# Unique Distribution of Diacyl-, Alkylacyl-, and Alkenylacyl-Phosphatidylcholine Species Visualized in Pork Chop Tissues by Matrix-Assisted Laser Desorption/Ionization–Mass Spectrometry Imaging

**DOI:** 10.3390/foods9020205

**Published:** 2020-02-16

**Authors:** Hirofumi Enomoto, Tomohiro Furukawa, Shiro Takeda, Hajime Hatta, Nobuhiro Zaima

**Affiliations:** 1 Department of Biosciences, Faculty of Science and Engineering, Teikyo University, Utsunomiya 320-8551, Japan; 2 Division of Integrated Science and Engineering, Graduate School of Science and Engineering, Teikyo University, Utsunomiya 320-8551, Japan; 3 Advanced Instrumental Analysis Center, Teikyo University, Utsunomiya 320-8551, Japan; 4 Department of Animal Science and Biotechnology, School of Veterinary Medicine, Azabu University, Sagamihara 252-5201, Japan; s-takeda@azabu-u.ac.jp; 5 Department of Food and Nutrition, Faculty of Home Economics, Kyoto Women’s University, Kyoto 605-8501, Japan; hatta@kyoto-wu.ac.jp; 6 Department of Applied Biological Chemistry, Graduate School of Agriculture, Kindai University, Nara 631-8505, Japan; zaima@nara.kindai.ac.jp; 7 Agricultural Technology and Innovation Research Institute, Kindai University, Nara 631-8505, Japan

**Keywords:** pork, phosphatidylcholines, lipid distribution, mass spectrometry imaging (MSI), matrix-assisted laser desorption/ionization (MALDI), liquid chromatography-tandem mass spectrometry (LC-MS/MS)

## Abstract

Phosphatidylcholine (PC) is the major phospholipid in meat and influences meat qualities, such as healthiness. PC is classified into three groups based on the bond at the *sn-1* position: Diacyl, alkylacyl, and alkenylacyl. To investigate their composition and distribution in pork tissues, including *longissimus thoracis et lumborum* (loin) spinalis muscles, intermuscular fat, and transparent tissues, we performed matrix-assisted laser desorption/ionization–mass spectrometry imaging (MALDI–MSI). Eleven diacyl-, seven alkylacyl-, and six alkenylacyl-PCs were identified using liquid chromatography (LC)-tandem MS (MS/MS) analysis. Despite many alkylacyl- and alkenylacyl-PC species sharing identical *m*/*z* values, we were able to visualize these PC species using MALDI–MSI. Diacyl- and alkylacyl- and/or alkenylacyl-PC species showed unique distribution patterns in the tissues, suggesting that their distribution patterns were dependent on their fatty acid compositions. PCs are a major dietary source of choline in meat, and the amount was significantly higher in the muscle tissues. Consumption of choline mitigates age-related memory decline and neurodegenerative diseases; therefore, the consumption of pork muscle tissues could help to mitigate these diseases. These results support the use of MALDI–MSI analysis for assessing the association between PC species and the quality parameters of meat.

## 1. Introduction

Consumer choice regarding meat consumption is influenced by the quality of the product, which is generally described by four parameters: Satisfaction, serviceability, security, and healthiness [1,2,3]. Healthiness, or nutritional quality, has become increasingly important due to a growing knowledge of how food components affect our physical and mental well-being. Meats, such as pork, are an important nutritional source, containing a variety of lipids, proteins, vitamins, and micronutrients. The most abundant phospholipids in meat are phosphatidylcholines (PCs) [4], which are components of the bilayer cell membrane and also function as a reservoir for polyunsaturated fatty acids (PUFAs). PUFAs are reported to have various health-promoting effects for people suffering from chronic diseases [5]. Choline, a key component of PCs, is both an essential micronutrient needed for proper organ function and a neuroprotectant required for normal development and growth of the fetal brain [6]. Owing to its popularity, pork represents a major dietary source of choline [7].

**Figure 1:**
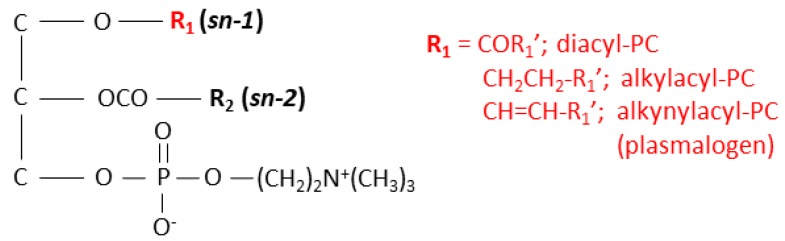
Structures of diacyl-, alkylacyl-, and alkenylacyl-phosphatidylcholine (PC) species.

PC comprises a glycerol backbone with fatty acids at the *sn-1* and *sn-2* positions and a phosphocholine head group at the *sn-3* position (Figure 1). The *sn-1* position of PC can be functionalized through acyl, ether, or vinyl-ether bonds and are subclassified on this basis as diacyl-, alkylacyl-(plasmanyl-), or alkenylacyl-(plasmenyl-/plasmalogen-) PCs, respectively [8,9,10]. The balance of these three classes of PC species differs among various organs and the PCs themselves are considered contributing factors to the quality, e.g., healthiness, of the meat. For example, plasmalogen-PC species have been reported to function as endogenous antioxidants [11,12], as well as lipid mediators. Studies also suggest that plasmalogens are involved in some chronic diseases [11,12]. Due to the important role of these biomolecules in human health, understanding their localization in meat sources is valuable for discerning which part of an animal is most nutritious. However, to date, the spatial distribution of PCs in pork meat is not fully understood.

Modern separation techniques, such as liquid chromatography (LC)–electrospray ionization (ESI)–mass spectrometry (MS), are used for the routine analysis of metabolites [4,7,13,14,15]. LC–ESI–MS can analyze nearly all lipid classes with high sensitivity and without major fragmentation. These techniques are useful for the qualitative and quantitative analysis of phospholipids and for investigating their spatial distribution by dividing samples into different tissues; however, the spatial resolution is dependent on the fineness of tissue differentiation. To resolve this problem, an emerging technique, mass spectrometry imaging (MSI), can be used to simultaneously investigate the content and spatial distribution of metabolites at microscopic resolution without the need for antibodies, staining, or complicated preliminary procedures [16,17,18,19,20,21,22,23,24,25,26,27,28,29,30,31,32,33]. MSI has been successfully used to separate multiple lipids according to their mass-to-charge ratio (*m*/*z*) and visualize the tissue distribution of each molecule [14,23,31,32,33]. Additionally, the use of tandem MS (MS/MS) allows the structure of the lipid components, i.e., types and fatty acids, to be elucidated [14,23,31,32,33]. Recently, we demonstrated that sphingomyelin species display different distribution patterns among pork chop tissues using matrix-assisted laser desorption/ionization (MALDI)-MSI [33].

In the present study, we sought to identify diacyl-, alkylacyl-, and alkenylacyl-PC species in pork chop using LC–ESI–MS/MS and MALDI–MS/MS analysis. Using MALDI-MSI, we attempted to visualize these PC species and showed their unique distribution and composition among four different tissues of pork chop. Our findings show that the coupling of MALDI-MSI with LC-ESI-MS/MS analysis is a useful tool for examining the nutritional qualities of meat tissues.

## 2. Materials and Methods 

### 2.1. Reagents

Water, methanol, potassium acetate, and formic acid were purchased from FUJIFILM Wako Pure Chemical Corp. (Tokyo, Japan). In addition, 2,5-dihydroxybenzoic acid (DHB) and α-cyano-4-hydroxycinnamic acid (CHCA) were purchased from Tokyo Kasei Kogyo Co., Ltd. (Tokyo, Japan). Peptide calibration standards, namely bradykinin (1–7) and angiotensin II, were purchased from Bruker Corp. (Billerica, MA, USA). All reagents and solvents used in the study were of analytical grade.

### 2.2. Pork Samples

Pork chops (six-month-old female crossbred pigs, Duroc, Danish Landrace, and Yorkshire) were purchased from a local supermarket one day after slaughter. The samples were stored in a freezer (−80 °C) until use.

### 2.3. Preparation of Pork Sections

Pork sections were prepared as described in our previous study [33]. Briefly, consecutive 10 μm sections containing four different tissues, namely loin, intermuscular fat tissue, transparent tissue, and spinalis muscle, were prepared using a cryostat (CRYOCUT CM1860; Leica Microsystems, Wetzlar, Germany). The frozen sections were mounted on indium tin oxide (ITO)-coated glass slides (100 Ω/m^2^ without MAS coating; Matsunami Glass, Osaka, Japan). The ITO-coated glass slides were put in 50 mL centrifuge tubes containing silica gel and preserved at −80 °C until MALDI–MSI analysis.

### 2.4. MALDI–MSI and MS/MS Analysis

MALDI–MSI analysis was performed as described in our previous study [33]. Briefly, a 40 mg/mL DHB solution with 20 mM potassium acetate in 70% aqueous methanol (1 mL) was sprayed uniformly over the pork sections using a 0.18 mm nozzle caliber airbrush (Mr. Airbrush Custom Double Action; Mr. Hobby, Tokyo, Japan). The sections were applied to a MALDI-TOF/TOF instrument (UltrafleXtreme, Bruker) equipped with a 355 nm Nd:YAG laser at a repetition rate of 1000 Hz. Data were collected in positive ion mode (reflector mode) at a step size of 100 μm. The laser diameter was set to medium size. *m*/*z* values ranging from 740 to 860 were measured and calibrated externally using the exact *m*/*z* of both bradykinin (1–7) [M + H]^+^ (*m*/*z* 757.39916) and angiotensin II [M + H]^+^ ions (*m*/*z* 1046.54180). All the spectra were collected automatically. Normalization was conducted based on total ion current using FlexImaging 4.1 software (Bruker). Two-dimensional ion-density maps were also prepared using this software.

Three sections prepared from the same pork chop were measured and the detection intensities in the loin, intermuscular fat tissue, transparent tissue, and spinalis muscle were extracted using the ‘region of interest’ function of FlexImaging 4.1 software. The detection intensities for identified PC species among each tissue were compared.

MALDI-MS/MS analyses were conducted using an UltrafleXtreme in collision-induced dissociation “LIFT” MS/MS mode, and selected precursor and product ions were acquired. The MS/MS spectra were analyzed using FlexAnalysis 3.4 software (Bruker).

### 2.5. Preparation of Total Lipid Extracts

Consecutive 10 μm pork chop sections (100 mg) were collected in microtubes. Total lipid extracts were prepared according to the Bligh and Dyer method [34]. After drying using a vacuum centrifugal evaporator (CVE-2200, Eyela Tokyo Rikakikai Co. Ltd., Tokyo, Japan), lipids were dissolved in 1 mL of methanol:acetonitrile:water (9:9:2, *v*/*v*/*v*) and determined by LC–ESI–MS/MS.

### 2.6. LC–ESI–MS/MS Analysis

LC–ESI–MS/MS analysis was performed as previously described [33] with some modifications. One microliter of total lipid extract was injected into an Agilent 1200 high-performance LC connected to an Agilent 6430 triple quadrupole mass spectrometer equipped with an ESI ion source (Agilent Technologies, Palo Alto, CA, USA). An Agilent Poroshell 120 EC-C18 reversed-phase column (100 × 2.1 mm, 2.7 μm particle size) was used at 40 °C. The mobile phase consisted of solvent A (acetonitrile:methanol:water (9:9:2, *v*/*v*/*v*)) and solvent B (isopropanol), both containing 5 mM ammonium formate. The LC flow rate was 0.2 mL/min. The LC gradient conditions were as follows: 0% B from 0 to 5 min, 0%–55% B from 5 to 60 min, 80% B from 60 to 80 min, and 0% B from 80 to 100 min. The MS instrument was set to positive ion mode, and the drying gas temperature was 300 °C (5.0 L/min). Precursor ion scanning at *m*/*z* 184.1, corresponding to the ionized polar head group, i.e., the phosphocholine [M + H]^+^ ion, was used to monitor the PC species. PC [M + H]^+^ precursor ions were analyzed using product ion scanning to identify the PC molecular species. The collision energy was set to 30 eV. Data analysis was performed using MassHunter software (Agilent Technologies).

### 2.7. Statistical Analyses

Data were expressed as mean values ± standard deviation (SD, *n* = 3). Statistical analyses were performed using GraphPad Prism 8.3.1 (GraphPad Software, San Diego, CA, USA). The data were compared using Welch’s ANOVA and the post-hoc analysis was performed using Dunnett’s T3 multiple comparisons test. Mean values with different letters indicate significant differences (*p* < 0.05). 

## 3. Results

### 3.1. Mass Spectrum Obtained from a Section of Pork Chop 

**Figure 2:**
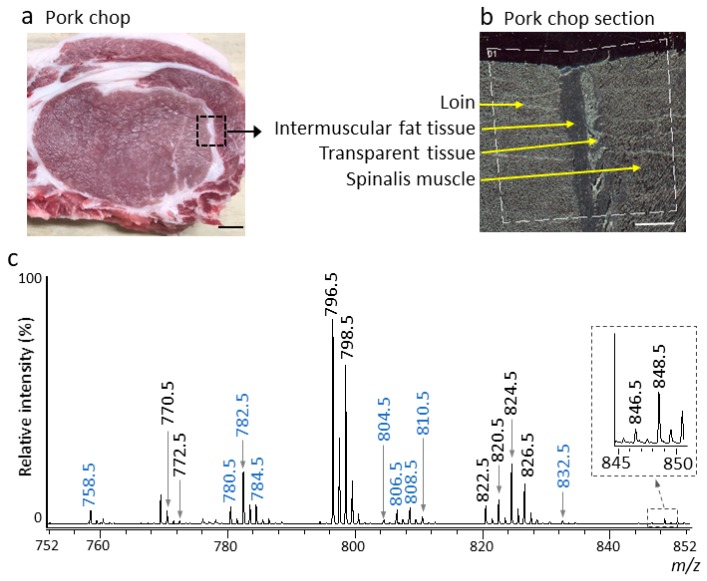
Matrix-assisted laser desorption/ionization–mass spectrometry imaging (MALDI-MSI) analysis of pork chop section. (**a**) Optical image of the pork chop used in the study. Loin, *Longissimus thoracis et lumborum* muscle. The dotted square line marks the analyzed region. Scale bar represents 10 mm. (**b**) Optical image of pork section prior to MALDI-MSI analysis. Scale bar represents 2 mm. (**c**) The mass spectrum at *m*/*z* 752 to 852. Peaks with black *m*/*z* values indicate diacyl-phosphatidylcholine (PC) species, while peaks with blue *m*/*z* values indicate alkylacyl- and/or alkenylacyl-PC species.

A portion of the pork chop containing four different tissues, loin, intermuscular fat tissue, transparent tissue, and spinalis muscle, was chosen for analysis by MALDI–MSI (Figure 2a,b). The spectrum of the pork chop was obtained in positive ion mode (Figure 2c). Phospholipids with phosphocholine, such as diacyl-PC and sphingomyelin species, have been reported to be selectively detected in animal tissues using DHB as a matrix [14,31,32,33]. We, therefore, used DHB as the matrix to analyze the diacyl-PC species and supplemented this with potassium acetate to detect these species as [M + K]^+^ ions [31,33]. Interestingly, along with the expected peaks for the diacyl-PC [M + K]^+^ ions, peaks with *m*/*z* differences of −16 or −14 were observed, e.g., *m*/*z* 780.5 and 782.5 for 796.5, or *m*/*z* 808.5 and 810.5 for 824.5, (Figure 2c). The *m*/*z* values of alkylacyl- and alkenylacyl-PC species are expected to decrease to −14 and −16, respectively (Figure 1), suggesting that alkylacyl- and alkenylacyl-PC [M + K]^+^ ions were also detected in the pork chop section. This is particularly notable as LC-ESI-MS/MS analysis of pork has generally only detected diacyl-PC species [4].

### 3.2. Identification of PC Species in Pork Chop Section

**Figure 3:**
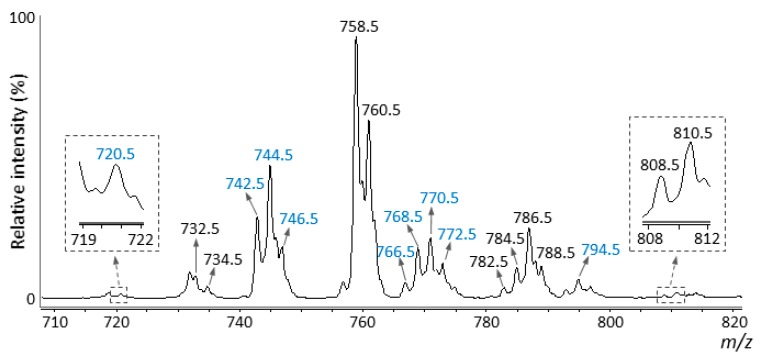
Mass spectrum obtained from the total lipid extract of pork chop sections using LC-MS/MS. Precursor ion scanning at *m*/*z* 184.1 in positive ion mode was performed. Peaks with black *m*/*z* values indicate diacyl-phosphatidylcholine (PC) species, while those with blue *m*/*z* values indicate alkylacyl- and/or alkenylacyl-PC species. All species were detected as [M + H]^+^ ions.

To identify diacyl-, alkylacyl-, and alkenylacyl-PC species in the pork chop, total lipid extracts were analyzed by precursor ion scanning at *m*/*z* 184.1 (phosphocholine head group) to detect phospholipid species, such as PC (Figure 1) and sphingomyelin species, selectively using LC–ESI–MS/MS [14,33]. PC species are detected as even numbers, while sphingomyelin species are detected as odd numbers, as dictated by the nitrogen rule [35]. All detected precursor ions with even numbers were further analyzed by product ion scanning to investigate the bond type at position *sn-1* and their fatty acid composition. The mass spectrum obtained from the total lipid extract by LC-ESI-MS/MS (Figure 3) showed a similar pattern to that obtained by MALDI-MSI (Figure 2c). In general, PC species have palmitic acid (16:0), stearic acid (18:0), and oleic acid (18:1) at the *sn-1* position and various fatty acids, including PUFA, at the *sn-2* position [8,9]. Based on the detected product ions from product ion scanning and previously published reports [4,13,14], eleven diacyl-, seven alkylacyl-, and six alkenylacyl-PCs were identified (Table 1). The relative intensities indicate the relative amounts of each PC species. Diacyl-PC species comprise 60.34% of the total composition, with the remaining 39.66% composed of alkylacyl- and/or alkenylacyl-PC species (Table 1). By contrast, the relative intensities of diacyl-PC species containing arachidonic acid (20:4) were lower than those of alkylacyl- and alkenylacyl-PC species containing arachidonic acid (Table 1).

PC species were detected as [M + H]^+^ ions using LC-ESI-MS/MS. The *m*/*z* values of the corresponding PC species [M + K]^+^ ions increased by 38 using MALDI-MSI. Based on the *m*/*z* values, PC species in the MALDI-MSI mass spectrum were assigned (Table 2). In addition, the presence of the phosphocholine head group was confirmed for the product ions in the MALDI-MS/MS spectra through the M-59 ions, which indicate [PC species – trimethylamine + K]^+^ ions and *m*/*z* 163, which is indicative of the [monoethyl phosphate + K]^+^ ion [14,23,31,32]. Peaks corresponding to all PC species identified by LC-ESI-MS/MS analysis were detected in the MALDI-MSI mass spectrum. However, PC (diacyl 16:0/16:1) could not be assigned, because the *m*/*z* value at 770.5 overlaps the *m*/*z* value of the isotope ion derived from the sphingomyelin (d18:1/18:0) monoisotopic ion (*m*/*z* 769.5) [33]. For the MALDI-MS/MS analysis, specific product ions used to distinguish different PC species with identical *m*/*z* values, e.g., *m*/*z* 824.5 of PC (diacyl 18:0/18:2) and PC (diacyl 18:1/18:1) [M + K]^+^ ions, or *m*/*z* 782.5 of PC (alkylacyl 16:0/18:2) and PC (alkenylacyl 16:0/18:1) [M + K]^+^ ions, were barely detected in the MS/MS spectra under our current experimental conditions, i.e., particular mass spectrometer and previously reported sample preparation methods [31]. In addition to *m*/*z* 824.5 and 782.5, *m*/*z* 806.5 and 810.5 also contained several different PC species (Table 2). The relative amounts of each PC species, as well as the relative composition of diacyl-PC species and alkylacyl- and/or alkenylacyl-PC species, showed similar patterns to those observed in LC-ESI-MS/MS analysis (Table 1).

### 3.3. Distribution of Diacyl-PC Species in Pork Chop

**Figure 4:**
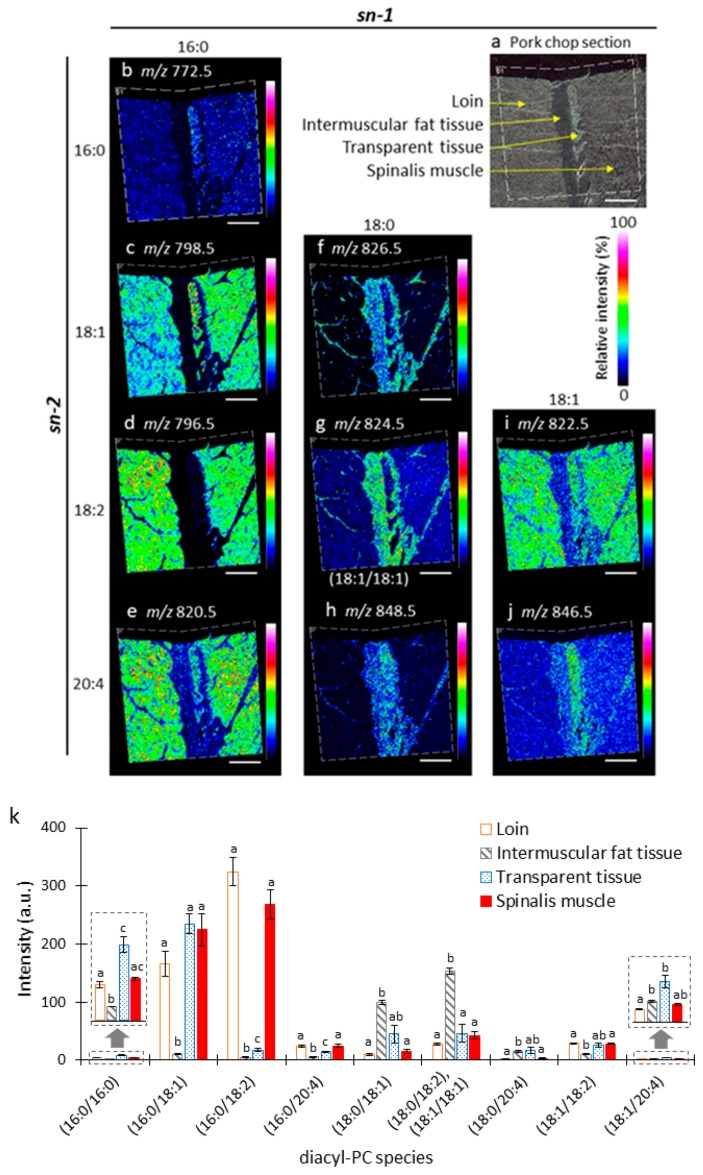
MALDI–MSI analysis of diacyl-phosphatidylcholine (PC) species in pork chop sections. (**a**) Optical image of pork chop section prior to MALDI–MSI analysis. (**b**–**j**) Representative ion images arranged according to their fatty acid compositions. Fatty acid compositions at the *sn-1* position are arranged along the x-axis, and those at the *sn-2* position are arranged along the y-axis. Scale bar represents 2 mm. (**k**) Intensities of diacyl-PC species in *longissimus thoracis et lumborum muscle* (loin), intermuscular fat tissue, transparent tissue, and spinalis muscle using MALDI-MSI. The data are expressed as the mean ± standard deviation (*n* = 3). Mean values with different letters indicate significant differences (*p* < 0.05) as determined by Dunnett’s T3 multiple comparisons test.

To investigate the distribution and composition of diacyl-PC species in loin, intermuscular fat tissue, transparent tissue, and spinalis muscle of the pork chop section, we reconstructed ion images from their *m*/*z* values (Figure 4b–j). The color gradient of the ion images shows the relative intensities of signals detected for each section. Three sections prepared from the same pork chop were analyzed, and the mean detection intensities of diacyl-PC species from each tissue were calculated (Figure 4k). The ion image of *m*/*z* 824.5 is composed of mixed ion images of PC (diacyl 18:0/18:2) and PC (diacyl 18:1/18:1) because they were detected as the same *m*/*z* value (Figure 4g). Interestingly, PC species showed unique distribution patterns among these tissues. Diacyl-PCs containing 16:0 at the *sn*-1 position were distributed in the loin and spinalis muscle (Figure 4b–e,k). When these diacyl-PC species also contained 16:0, 18:1, and 20:4 at the *sn*-2 position, they were also located in the transparent tissue (Figure 4b,c,e,k). Diacyl-PC species containing 18:0 at the *sn*-1 position were distributed in the intramuscular fat tissue (Figure 4f–h,k). When these diacyl-PC species contained 20:4 at the *sn*-2 position, they were also distributed in the transparent tissue (Figure 4h,k). Diacyl-PC species containing 18:1 at the *sn*-1 position and linoleic acid (18:2) at the *sn*-2 position was found across loin, transparent tissue, and spinalis muscle (Figure 4i,k), whereas that containing 20:4 at the *sn*-2 position was enriched in the transparent tissue (Figure 4j–k). These results suggest that the distribution patterns of diacyl-PC species among these pork chop tissues are dependent on the fatty acid composition, particularly at *sn-1*.

### 3.4. Distribution of Alkylacyl- and/or Alkenylacyl-PC Species in Pork Chop

**Figure 5:**
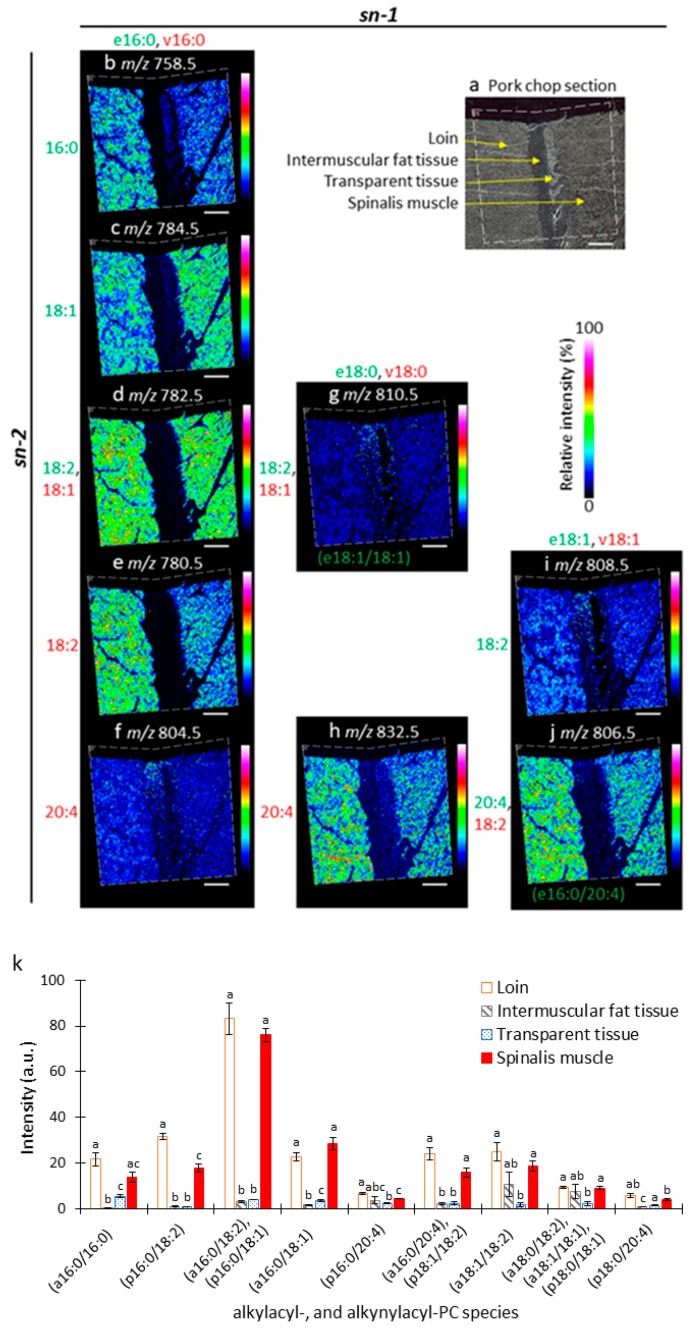
MALDI-MSI analysis of alkylacyl- and alkenylacyl-phosphatidylcholine (PC) species in pork chop sections. (**a**) Optical image of pork chop section prior to MALDI–MSI analysis. (**b**–**j**) Representative ion images arranged according to their fatty acid compositions. Fatty acid compositions at the *sn-1* position are arranged along the x-axis, and those at the *sn-2* position are arranged along the y-axis. In the *sn-1* position, ‘e’ and ‘v’ indicate ether and vinyl-ether bonds, respectively; therefore, green and red represent alkylacyl- or alkenylacyl-PC species, respectively. Scale bar represents 2 mm. (**k**) Intensities of alkylacyl- and alkenylacyl-PC species in *longissimus thoracis et lumborum* muscle (loin), intermuscular fat tissue, transparent tissue, and spinalis muscle using MALDI-MSI. The data are expressed as the mean ± standard deviation (*n* = 3). Mean values with different letters indicate significant differences (*p* < 0.05) as determined by Dunnett’s T3 multiple comparisons test.

To investigate the distribution and composition of alkylacyl- and/or alkenylacyl-PC species in loin, intermuscular fat tissue, transparent tissue, and spinalis muscle of the pork chop section, we reconstructed ion images using their *m*/*z* values (Figure 5b–j). The mean detection intensities of alkylacyl- and/or alkenylacyl-PC species from each tissue were calculated (Figure 5k). The ion images of *m*/*z* 782.5, 806.5, and 810.5 are the mixed ion images of PC (alkylacyl 16:0/18:2) and PC (alkenylacyl 16:0/18:1), PC (alkylacyl 16:0/20:4) and PC (alkenylacyl 18:1/18:2), or PC (alkylacyl 18:0/18:2), PC (alkylacyl 18:1/18:1), and PC (alkenylacyl 18:0/18:1), respectively, because they were detected as the same *m*/*z* value (Figure 5d,g,j). Similarly to diacyl-PCs, alkylacyl- and alkenylacyl-PC species showed unique distribution patterns among the tissues. All alkylacyl- and alkenylacyl-PC species were primarily distributed in the loin and spinalis muscle tissues (Figure 5b–k). Alkylacyl- and/or alkenylacyl-PC species containing 16:0 at the *sn*-1 position and 20:4 at the *sn-2* position (Figure 5f,k), 18:0 or 18:1 at the *sn-1* position and 18:1 or 18:2 at the *sn-2* position (Figure 5g,k), and 18:1 at the *sn-1* position and 18:2 at the *sn-2* position (Figure 5i,k) were also distributed in intermuscular fat tissue. These results suggest that distribution patterns of alkylacyl- and/or alkenylacyl-PC species among these pork chop tissues are also dependent on the fatty acid compositions.

### 3.5. Amounts of Diacyl-, Alkylacyl-, and Alkenylacyl-PC Species in Pork Chop

**Figure 6:**
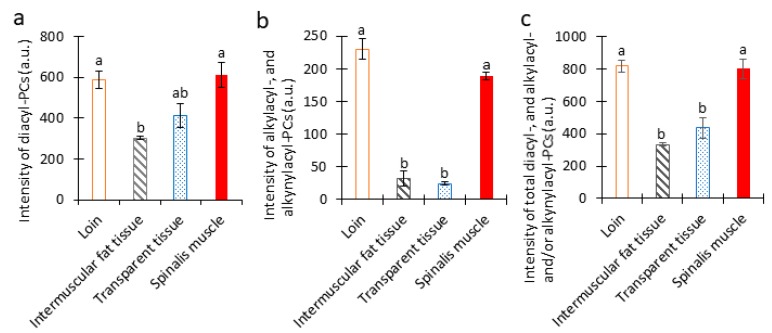
Amounts of total diacyl-, alkylacyl-, and alkenylacyl-phosphatidylcholine (PC) species in each tissue of a pork chop section. Intensities of (**a**) total diacyl-, (**b**) total alkylacyl- and alkenylacyl-PC species, and (**c**) total of all three PC species in *longissimus thoracis et lumborum* muscle (loin), intermuscular fat tissue, transparent tissue, and spinalis muscle using MALDI-MSI. The data are expressed as the mean ± standard deviation (*n* = 3). Mean values with different letters indicate significant differences (*p* < 0.05) as determined by Dunnett’s T3 multiple comparisons test.

To compare the amounts of the total diacyl-, alkylacyl-, and alkenylacyl-PC species within and between loin, intermuscular fat tissue, transparent tissue, and spinalis muscle, their intensities within each tissue were combined (Figure 6). The amounts of total diacyl-PC species were predominantly higher in loin and spinalis muscle than in transparent and intramuscular fat tissue (Figure 6a). The amounts of total alkylacyl- and/or alkenylacyl-PC species were also significantly higher in both muscles than in the other two tissues (Figure 6b). In fact, the disparity was even greater for these PC species than for the diacyl-PC species. The amounts of total diacyl-, alkylacyl- and/or alkenylacyl-PC species were significantly higher in both muscles than in the other two tissues (Figure 6c).

## 4. Discussion

In this study, we sought to identify and visualize diacyl-PC species, as well as alkylacyl- and alkenylacyl-PC species in pork chop using both LC–ESI–MS/MS and MALDI–MSI analyses. We identified eleven diacyl-, seven alkylacyl-, and six alkenylacyl-PC species using LC-ESI-MS/MS analysis and simultaneously visualized these species in pork sections. Their distribution and composition in loin, intramuscular fat tissue, transparent tissue, and spinalis muscle were comprehensively determined.

Diacyl-phospholipid or ether-linked phospholipids (alkylacyl- and alkenylacyl-phospholipids) are synthesized through a well-characterized process that begins in the peroxisome and is completed in the endoplasmic reticulum [10]. Balancing the action of peroxisomal enzymes—acyl-CoA synthetase (ACS) versus fatty acyl-CoA reductase (FAR1/2) and glyceronephosphate *O*-acyltransferase (GNPAT) versus alkylglycerone phosphate synthase (AGPS)—helps to regulate the relative amount of diacyl-phospholipid and ether-linked phospholipid synthesized [10]. The actions of ACS and GNPAT lead to the biosynthesis of diacyl-phospholipid, while those of FAR1/2 and AGPS lead to the biosynthesis of ether-linked phospholipids [10]. In this study, we demonstrated that the amounts of total diacyl-PC species and alkylacyl- and/or alkenylacyl-PC species were higher in both muscles than in the other two tissues, with a greater difference observed for alkylacyl- and/or alkenylacyl-PC species than for the diacyl-PC species (Figure 6a,b). Based on these results, we speculate that the actions of FAR1/2 and/or AGPS are lower in the intermuscular fat and transparent tissues than in both the muscle tissues.

In the processing of animal species such as pigs at slaughterhouses, by-products containing fat tissues are produced in yields ranging from 10% to 30% of the live weight [36]. In this study, we revealed that intermuscular fat tissue contains a relatively large amount of diacyl-PC species, especially those containing stearic acid (18:0) at the *sn*-1 position (Figure 4f–h,k). These diacyl-PC species also contain PUFAs, particularly 18:2 and 20:4 at the *sn*-2 position. Therefore, by-product fat is a potential food source of these diacyl-PC species and PUFAs.

The literature has reported that PC species contain the majority of choline in meat samples, comprising a mean of 82.6 ± 5.5% total choline [7]. In this study, we showed that the amount of total PC species was significantly higher in both muscle tissues than in the other two tissues (Figure 6c). Animal and human studies have shown that prenatal or perinatal supplementation of choline influences fetal health and vulnerability to disease later in life. For example, prenatal or perinatal supplementation of choline in rodents attenuated stress, modulated behavior, and improved memory and cognitive functions in the adult offspring [6]. Choline has also been shown to mitigate symptoms associated with genetically related neurodegenerative diseases, such as Alzheimer’s disease [6]. Therefore, consuming pork muscle tissues might mitigate age-related memory decline and neurodegenerative diseases.

In this study, we could not separately visualize alkylacyl- and alkenylacyl-PC species, because some of these have identical *m*/*z* values, and specific product ions were not detected in these experimental conditions. It is necessary to investigate the distribution of alkenylacyl-PC species further to estimate the healthiness of each tissue in pork, because plasmalogens may be involved in cancer or age-related neurodegenerative disorders, such as Alzheimer’s disease or Parkinson’s disease [11,12]. In addition, red meat containing pork has been reported as a potential cause of chronic diseases, such as colorectal cancer [3]. The literature has reported the utility of linear ion-trap multiple-stage mass spectrometry to analyze diacyl-, alkylacyl-, and alkenylacyl-PC species separately [9]. Therefore, linear ion-trap MALDI-MSI mass spectrometry might be capable of separately visualizing these three PC species.

## 5. Conclusions

We identified unique distribution patterns of diacyl-, alkylacyl-, and alkenylacyl-PC species in loin, intermuscular fat tissue, transparent tissue, and spinalis muscle in pork chop using a combination of MALDI-MSI and LC-ESI-MS/MS analysis. This analytical technique could contribute to assessing the qualities of various meats, such as healthiness, based on the content of PC species in each tissue.

## Figures and Tables

**Table 1:** Phosphatidylcholine (PC) species in pork chop detected by LC–electrospray ionization (ESI)–MS/MS.

[M + H]^+^, *m*/*z*	Molecular Species	Relative Intensity (%)	Product ions for Assignment, *m*/*z*
732.5	(diacyl 16:0/16:1)	1.20 ± 0.08	184, 476, 478
734.5	(diacyl 16:0/16:0)	1.08 ± 0.07	184, 478
758.5	(diacyl 16:0/18:2)	27.13 ± 0.96	184, 478, 502
760.5	(diacyl 16:0/18:1)	16.77 ± 0.51	184, 478, 504
782.6	(diacyl 16:0/20:4)	0.88 ± 0.07	184, 478, 526
784.6	(diacyl 18:1/18:2)	2.53 ± 0.07	184, 502, 504
786.6	(diacyl 18:0/18:2) (diacyl 18:1/18:1)	5.04 ± 0.09 2.34 ± 0.05	184, 502, 506 184, 504
788.6	(diacyl 18:0/18:1)	2.67 ± 0.10	184, 504, 506
808.6	(diacyl 18:1/20:4)	0.28 ± 0.03	184, 504, 526
810.6	(diacyl 18:0/20:4)	0.43 ± 0.01	184, 506, 526
	Total	60.34	
720.5	(alkylacyl 16:0/16:0)	0.48 ± 0.02	184, 482, 478
742.5	(alkenylacyl 16:0/18:2)	8.67 ± 0.21	184, 480, 502
744.5	(alkylacyl 16:0/18:2) (alkenylacyl 16:0/18:1)	11.40 ± 0.29 1.40 ± 0.06	184, 482, 502 184, 480, 504
746.5	(alkylacyl 16:0/18:1)	3.88 ± 0.20	184, 482, 504
766.6	(alkenylacyl 16:0/20:4)	1.50 ± 0.04	184, 480, 526
768.6	(alkylacyl 16:0/20:4) (alkenylacyl 18:1/18:2)	2.20 ± 0.12 2.13 ± 0.03	184, 482, 526 184, 506, 502
770.6	(alkylacyl 18:1/18:2)	3.59 ± 0.18	184, 502, 508
772.6	(alkylacyl 18:0/18:2) (alkylacyl 18:1/18:1) (alkenylacyl 18:0/18:1)	0.86 ± 0.04 0.97 ± 0.03 1.15 ± 0.01	184, 510, 502 184, 508, 502 184, 508, 504
794.6	(alkenylacyl 18:0/20:4)	1.43 ± 0.01	184, 508, 526
	Total	39.66	

The total lipid extracts from the pork chop sections were subjected to precursor ion scanning at *m*/*z* 184.1 to detect phosphatidylcholine (PC) species. Product ion scanning was used for the analysis of fatty acid composition. PC species were assigned based on product ions and previous reports [4,13,14]. The relative intensities of identified PC species are presented as their average ± standard deviation (*n* = 3).

**Table 2:** Phosphatidylcholine (PC) species in pork chop detected by MALDI-MS/MS.

[M + K]^+^, *m*/*z*	Molecular Species	Relative Intensity (%)	Product Ions for Assignment, *m*/*z*
772.5	(diacyl 16:0/16:0)	0.45 ± 0.03	163, 713
796.5	(diacyl 16:0/18:2)	31.21 ± 3.79	163, 737
798.5	(diacyl 16:0/18:1)	24.38 ± 2.77	163, 739
820.5	(diacyl 16:0/20:4)	2.87 ± 0.11	163, 761
822.5	(diacyl 18:1/18:2)	3.65 ± 0.07	163, 763
824.5	(diacyl 18:0/18:2), (diacyl 18:1/18:1)	8.59 ± 0.97	163, 765
826.5	(diacyl 18:0/18:1)	5.88 ± 0.60	163, 767
846.5	(diacyl 18:1/20:4)	0.24 ± 0.01	163, 787
848.5	(diacyl 18:0/20:4)	0.83 ± 0.06	163, 789
	Total	78.10	
758.5	(alkylacyl 16:0/16:0)	1.76 ± 0.16	163, 699
780.5	(alkenylacyl 16:0/18:2)	2.53 ± 0.08	163, 721
782.5	(alkylacyl 16:0/18:2) (alkenylacyl 16:0/18:1)	8.15 ± 0.86	163, 723
784.5	(alkylacyl 16:0/18:1)	2.88 ± 0.25	163, 725
804.6	(alkenylacyl 16:0/20:4)	0.59 ± 0.04	163, 745
806.6	(alkylacyl 16:0/20:4) (alkenylacyl 18:1/18:2)	2.01 ± 0.12	163, 747
808.6	(alkylacyl 18:1/18:2)	2.33 ± 0.11	163, 749
810.6	(alkylacyl 18:0/18:2) (alkylacyl 18:1/18:1) (alkenylacyl 18:0/18:1)	1.76 ± 0.16	163, 751
832.6	(alkenylacyl 18:0/20:4)	0.44 ± 0.04	163, 773
	Total	21.90	

MALDI-MS/MS analysis was performed directly on pork chop sections. Relative intensities of individual PC species are presented as mean ± standard deviation (*n* = 3).
